# Determining the Range for Appropriate Gestational Weight Gain Proportion for Singleton Pregnant Chinese Women

**DOI:** 10.1002/fsn3.70289

**Published:** 2025-05-27

**Authors:** Yin Jia, Haili Jiang, Yang Yu, Yuhui Fu, Yue Li, Huili Wang

**Affiliations:** ^1^ School of General Practice and Continuing Education Capital Medical University Beijing China; ^2^ Department of Obstetrics, Beijing Obstetrics and Gynecology Hospital Capital Medical University, Beijing Maternal and Child Health Care Hospital Beijing China; ^3^ School of Public Health Capital Medical University Beijing China

**Keywords:** adverse pregnancy outcomes, body mass index, gestational weight gain

## Abstract

To examine the optimal proportion of gestational weight gain (GWG) for Chinese singleton women based on Chinese body mass index (BMI) categories. A retrospective cohort study with 16,977 singleton pregnant women was conducted. Among the included subjects, 2/3 of whom were randomly imported into the training set for calculating the optimal proportion of GWG ranges, and another third of the women were imported into the validation set to evaluate the GWG ranges calculated with different methods. The detection rate of adverse outcomes of pregnant women with appropriate weight gain proportion under the above three methods was evaluated by paired chi‐square test, using the data of pregnant women in the validation database. According to the percentile method, the suitable proportion of GWG of pregnant women with underweight, normal weight, overweight, or obesity before pregnancy was 25.8%–37.8%, 20.3%–30.8%, 13.0%–23.1%, respectively. According to the Odds ratio method, the suitable proportion of GWG for pregnant women with different BMI categories was 24%–34%, 14%–18%, and 4%–14%, respectively. According to the combined risk curve method, the suitable proportion of GWG for pregnant women with different BMI categories was 27.9%–32.5%, 11.5%–16.8%, and −2.1%–5.5%, respectively. When the proportion of GWG ranges from 27.9%–32.5%, 11.5%–16.8%, and −2.1%–5.5% for different BMI categories, the pregnant women with appropriate weight gain proportions have the lowest detection rate of adverse maternal and infant outcomes. The GWG proportion serves as a valuable clinical indicator for enabling personalized weight management strategies and tailored nutritional guidance during pregnancy. The recommended proportion of GWG in this study for underweight, normal, overweight, and obese pregnant women was 27.9%–32.5%, 11.5%–16.8%, and −2.1%–5.5%, respectively.

## Graphical abstract

This study established gestational weight gain (GWG) proportion recommendations as follows: 27.9%–32.5% for underweight women, 11.5%–16.8% for normal weight women, and −2.1% to 5.5% for overweight/obese women. Within these recommended GWG ranges, pregnant women showed the lowest detection rates of adverse maternal and infant outcomes.

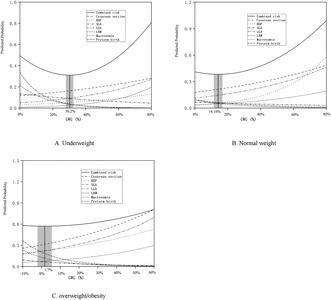

## 1 Background

Pregnancy requires a significant amount of nutrition and energy, and gestational weight gain (GWG) is the best parameter to appropriately gauge the nutritional status of pregnant women and infants. Previous reports have shown that excessive and rapid weight gain during pregnancy can significantly elevate the risk of complications and adverse outcomes such as pregnancy‐induced hypertension, pregnancy‐induced diabetes, postpartum obesity, and even long‐term metabolic diseases (including hypertension, obesity and diabetes) (Oken and Gillman 2003; Sun et al. 2020; Timmermans et al. 2020; Goldstein et al. 2018). On the other hand, insufficient GWG can cause intrauterine growth retardation and fetal growth restriction, leading to an elevated risk of adverse pregnancy‐related outcomes, including premature birth, low birth weight (LBW) of newborns, and even central obesity, hypertension, and hyperlipidemia later in life (Oken and Gillman 2003; Liew et al. 2008; Jaquet and Czernichow 2003; Tang et al. 2021).

Currently, the GWG guideline issued by the Institute of Medicine (IOM) in 2009 is the most authoritative and widely used recommendation globally (Rasmussen et al. 2009). However, the IOM 2009 guideline was originally formulated for the management of gestational weight of American women, and there may be certain differences between eastern and western pregnant women on account of variations in race, body conditions, economic status, geographical environment, eating habits, activity levels, psychological state, stress levels, as well as the criteria for classification of pre‐pregnancy body mass index (BMI) (Sun et al. 2020; Rosal et al. 2016; Titapant 2013). Therefore, the GWG guideline recommended by IOM in 2009 may not be suitable for pregnant women in China, making it necessary to establish the range for appropriate GWG based on maternal data in China.

The indicator presently used in most studies to measure GWG is the change in maternal weight (kg), that is, the difference between pre‐delivery and pre‐pregnancy weights. This indicator is simple and convenient for clinical use, but it is not appropriate to assess weight gain in pregnant women in an individualized and comprehensive manner. Besides weight change, the most commonly used weight management index is BMI (kg/m^2^). Unlike the weight change parameter that only considers body weight, BMI introduces the height variable, which can more accurately reflect a person's obesity and thinness. Indeed, some previous studies on GWG have used BMI change as a metric (Xiong et al. 1998; Paljk et al. 2021). However, because BMI calculations can be tedious and inconvenient for clinical application, BMI changes during pregnancy have not been used for weight management during pregnancy.

As such, compared with the parameter of change in maternal body weight, the proportion of body weight change (referred to as GWG proportion in this study; %), that is, GWG (kg) divided by pre‐pregnancy weight (kg), may be a more accurate metric to reflect the degree of weight change of women during pregnancy. Furthermore, this metric may be more suitable for evaluating the link between GWG and adverse pregnancy and neonatal outcomes.

The objective of the current study was to identify the ranges of appropriate GWG proportion for Chinese women based on the combined risks of pregnancy and neonatal outcomes, so the risk of these adverse outcomes can be minimized and women's health can be promoted further.

## 2 Methods

### 2.1 Study Population

**FIGURE 1:**
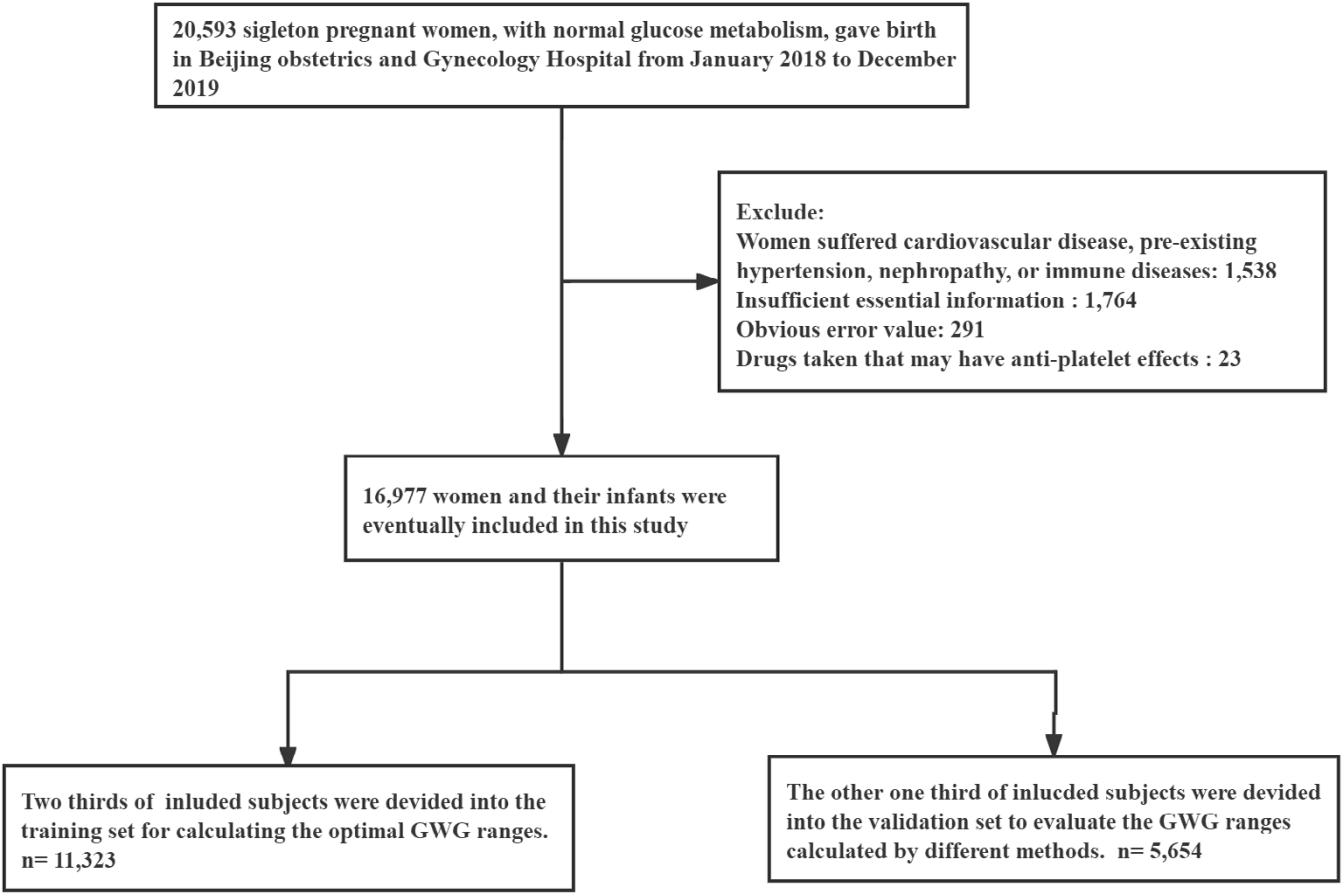
Flowchart depicting the implementation of the inclusion and exclusion criteria on the research population.

The present study focused on pregnant women who were singleton and had given birth at the Beijing Obstetrics and Gynecology Hospital between the months of January 2018 and December 2019. This hospital is the largest of its kind in North China and delivers about one‐tenth of all newborns in Beijing each year. The following inclusion criteria were used: (1) subjects had obtained their prenatal care and delivered their babies at the hospital; (2) they were in good health without a history of hypertension, blood disorders, cardiovascular disease, or diabetes; (3) they did not have a family history of diabetes or hypertension; (4) they gave birth to a single live baby. Additionally, the following exclusion criteria were implemented: (1) cases with lethal fetal malformations; (2) stillbirths; (3) diagnoses of gestational diabetes mellitus; (4) missing data on pre‐pregnancy weight, height, or weight before delivery; (5) incomplete information on Apgar score, birth length, birth weight, or gestational age. The detailed process of selecting and excluding research subjects can be found in Figure 1.

### 2.2 Study Design and Grouping

Using SPSS, a column of random numbers was generated with a fixed seed value (“1234567890”) to ensure reproducibility. The dataset was then sorted in ascending order. The first 11,323 cases were assigned to the training set for estimating the appropriate ranges of GWG proportions. The remaining 5654 cases (from case 11,324 to 16,977) were designated as the validation set to assess the GWG ranges derived from different analytical approaches. Pre‐pregnancy BMI was categorized according to the criteria recommended for Chinese adults by the Working Group on Obesity in China (WGOC) (Chen et al. 2004).

This study has been approved by the appropriate ethics committee of Beijing Obstetrics and Gynecology Hospital and has therefore been performed in accordance with the ethical standards laid down in the 1964 Declaration of Helsinki and its later amendments. The approval number is 2022‐KY‐019‐01. All persons gave their verbal informed consent prior to their inclusion in the study.

### 2.3 Anthropometric Measurements

All relevant data were procured through the electronic healthcare record system of the Beijing Maternity Hospital (affiliated to the Capital Medical University). The baseline data collected for each subject included the following metrics: Age of pregnant women, weight before pregnancy (self‐reported), height, gestational weeks of delivery, parity, birth weight, mode of delivery, and gender of newborns. Pregnancy complications and adverse pregnancy outcomes included hypertensive disorder complicating pregnancy, pre‐eclampsia, all‐cause cesarean section, cesarean section with medical indications, LBW, small for gestational age (SGA), large for gestational age (LGA), macrosomia, premature delivery, postpartum hemorrhage, and neonatal asphyxia. Note that pre‐pregnancy BMI and GWG proportion were obtained using these formulae:

$$\text{Pre‐pregnancy}\;\text{BMI}=\text{pre‐pregnancy weight}\;\left(\text{kg}\right)/{\left[\text{height}\;\left(m\right)\right]}^{2}$$

$$\begin{array}{c} \text{GWG}\;\text{proportion}=\left(\text{pre‐delivery weight}\;\left(\text{kg}\right)–\text{pre‐pregnancy weight}\;\left(\text{kg}\right)\right)/\\ \text{pre‐pregnancy weight}\;\left(\text{kg}\right)\times 100\%. \end{array}$$

The number of gestational weeks of delivery was calculated by a competent doctor using the time interval from the first day of the last menstrual cycle of the subject to the time of delivery. The weight before delivery (in kg) was measured by a midwife before the parturient entered the delivery room. The weight of the newborn (in g) was measured within 1 h of birth by the midwife in the delivery room with a baby scale. Similarly, the length of the newborn (in cm) was measured within 1 h of birth by the midwife in the delivery room with a soft ruler. The indications of cesarean section were also recorded by the midwife after consulting all the cases, which were divided into three categories, namely, cesarean section with medical indications, cesarean section with social factors, and cesarean section after trial of labor.

### 2.4 Definition of Outcomes

Adverse maternal and infant outcomes included cesarean section with medical indications, hypertensive disorder complicating pregnancy, SGA infants, LGA infants, LBW infants, macrosomia, and premature birth. Composite endpoints included one or more of the above adverse maternal and neonatal outcomes.

The medical indications of cesarean section of each pregnant woman were recorded by the midwife by referring to the individual case. Hypertensive disorder complicating pregnancy (HDP) included hypertension cases diagnosed before pregnancy or new cases of hypertension diagnosed during the gestational stage (Yundai et al. 2019). Pre‐eclampsia was characterized by hypertension and proteinuria in the second half of pregnancy, which subsides soon after delivery (Filipek and Jurewicz 2018). Preterm birth was defined as delivery occurring before 37 completed weeks of gestation (WHO 1977). LBW was classified as a live‐born neonate weighing less than 2500 g at birth (Hughes et al. 2017), while macrosomia referred to a live birth with a birth weight exceeding 4000 g (Araujo Júnior et al. 2017). SGA was defined as a birth weight below the 10th percentile for neonates of the same sex and gestational age. LGA was defined as a birth weight above the 90th percentile based on sex and gestational age‐specific standards (Capital Institute of Pediatrics and Nine‐City Joint Investigation Group of Children's Physical Development 2020). Neonatal asphyxia was identified based on an Apgar score less than 8 in 1 min or 5 min after birth (Solevåg et al. 2019). Postpartum hemorrhage was defined as blood loss exceeding 500 mL following vaginal delivery or more than 1000 mL following cesarean section, occurring within the first 24 h after childbirth (Evensen et al. 2017).

### 2.5 Statistical Analysis

The percentile method is a common method reported in previous studies (Wu et al. 2019; Wang et al. 2018). The range for appropriate GWG proportion was determined as the interval between the 25th and the 75th percentile of GWG proportion of pregnant women with no occurrence of composite endpoint events in the training set.

The odds ratio (OR) method was previously used by Cedergren et al. to analyze appropriate GWG in Sweden in 2007 (Cedergren 2007). In accordance with previously reported methods to determine appropriate GWG, pregnant women in different BMI groups were grouped into different weight gain classes with every 2% weight gain, and those with only a few people in some weight gain intervals were grouped into one group (Hytten 1990). The risk of adverse outcomes in each weight gain class was compared with those in all other weight gain classes in the same BMI group. A multivariate logistic regression analysis was conducted to calculate the odds ratios (ORs) and 95% confidence intervals (CIs) of adverse pregnant outcomes, after adjusting for maternal age, pre‐pregnancy BMIs, gestational weeks of delivery, and parity. The establishment of the optimal GWG range was based on weight gain intervals corresponding to OR values less than 1 and the upper and lower cutoff values of 95% CIs.

Based on previous studies (Beyerlein et al. 2009; Choi et al. 2017), the combined risk curve method was also used to analyze the appropriate GWG. This method was optimized as per the actual clinical situation of Chinese pregnant women. Meanwhile, the exponential function model was applied in order to improve the fitness between GWG and the predicted probability of a single adverse outcome. On the other hand, the quadratic function model was used to improve the fitness between GWG and the total predicted probability. The range for appropriate GWG proportion identified by this method corresponds to the lowest combined risk increase of no more than 1‰.

The baseline characteristics and the incidence of adverse pregnancy‐related outcomes in the training and validation sets were compared. The t‐test was employed to statistically analyze continuous variables, while the chi‐squared (*χ* ^2^) test was used for categorical variables. Three analytical methods—the percentile method, the disease risk method, and the combined risk curve method—were applied to the training dataset to determine the appropriate ranges for GWG proportion. Subsequently, the three GWG proportion ranges derived from these methods were evaluated using paired *χ* ^2^ tests, based on data from pregnant women in the validation set.

All data were recorded using Epidata 3.0, while statistical analyses were conducted using the SPSS 22.0 software. After a normality test of the continuous variables, measures conforming to a normal distribution were presented as mean ± standard deviation ($\bar{x}$ ± s), while others were presented as median and interquartile range. The count data were statistically described as frequency (*n*) and percentages (%). All continuous variables were analyzed using analysis of variance (ANOVA), while the categorical variables were statistically compared using the chi‐squared test. A *p*‐value < 0.05 was used to identify statistically significant differences.

## 3 Results

This study finally included 16,977 pregnant women. The flow chart of inclusion and exclusion of research subjects is presented in Figure 1.

### 3.1 Basic Characteristics of the Study Subjects

The mean age of the study subjects was found to be 32.0 ± 3.8 years and the mean GWG proportion was 25.6% ± 9.6%. The rate of nulliparity was 12,172 (71.7%). Among the 16,977 newborns, 8771 (51.7%) were boys and 8206 (48.3%) were girls. The mean birth weight of included infants was 3343.3 ± 480.4 g and the mean birth height was 50.0 ± 2.0 cm. According to the BMI classification standard recommended by the WGOC (Chen et al. 2004), 2298 (13.5%) of the subjects were underweight, 11,379 (67.0%) were at normal weight, and 3300 (19.4%) were overweight or obese before pregnancy. The baseline characteristics of the study subjects are described in Table 1.

**TABLE 1:** Basic characteristics of the included mother–infant pairs (*n* = 16,977).

Variables	$\bar{x}$ ± *s*/*n* (%)
Mother's age (years), mean ± SD	32.0 ± 3.8
Mother's height (cm), mean ± SD	163.0 ± 5.0
Pre‐pregnancy weight (kg), mean ± SD	57.5 ± 8.9
Mother's pre‐pregnancy BMI (kg/m^2^), mean ± SD	21.64 ± 3.13
Gestational weight gain proportion (%), mean ± SD	25.6 ± 9.6
Gestational week (weeks), mean ± SD	38.9 ± 1.7
Pre‐pregnancy BMI classification, *n* (%)	
Underweight (< 18.5 kg/m^2^)	2298 (13.5%)
Normal weight (18.5–23.9 kg/m^2^)	11,379 (67.0%)
Overweight (25.0–27.9 kg/m^2^)	2578 (15.2%)
Obesity (≥ 28.0 kg/m^2^)	722 (4.3%)
Parity, *n* (%)	
Primiparity	12,172 (71.7%)
Multiparity	4805 (28.3%)
Gender of newborn, *n* (%)	
Girl	8206 (48.3%)
Boy	8771 (51.7%)
Neonatal length (cm), mean ± SD	50.0 ± 2.0
Neonatal weight (g), mean ± SD	3343.3 ± 480.4

This study adopts the research design of training and validation sets. Specifically, the study subjects were randomly distributed between the two sets: 66.7% (11,323 cases) were included in the training set, while the rest (5654 cases) were included in the validation set. The baseline characteristics of the two sets showed no obvious differences, and the incidence of adverse maternal and neonatal outcomes in the two sets also did not show any statistically significant differences (Table 2).

**TABLE 2:** Comparison of basic characteristics and outcomes between two data sets.

Variables	Training set (*n* = 11,323)	Validation set (*n* = 5654)	*t*/*χ* ^2^	*p*
Mother's age (years), mean ± SD	32.0 ± 3.9	32.1 ± 3.8	−0.369	0.712
Mother's height (cm), mean ± SD	163.0 ± 5.0	163.0 ± 5.0	0.012	0.991
Pre‐pregnancy BMI (kg/m^2^), mean ± SD	21.7 ± 3.2	21.6 ± 3.1	1.504	0.133
Gestational weight gain (%), mean ± SD	25.5% ± 9.6%	25.6% ± 9.7%	−0.790	0.430
Gestational week (weeks), mean ± SD	38.9 ± 1.7	38.9 ± 1.7	−0.578	0.563
Neonatal weight (g), mean ± SD	3340.2 ± 480.4	3349.4 ± 480.4	−1.171	0.471
Neonatal length (cm), mean ± SD	50.0 ± 2.0	50.0 ± 2.0	−0.727	0.977
Primiparity, *n* (%)	8142 (71.9%)	4030 (71.3%)	0.737	0.391
Cesarean delivery, *n* (%)	3392 (30.0%)	1761 (31.1%)	2.523	0.112
CS with medical indications, *n* (%)	2379 (21.1%)	1206 (21.3%)	0.231	0.631
Pre‐eclampsia, *n* (%)	545 (4.8%)	262 (4.6%)	0.268	0.605
Postpartum hemorrhage, *n* (%)	663 (5.9%)	314 (5.6%)	0.633	0.426
Gender of newborn (boy), *n* (%)	5855 (51.7%)	2916 (51.6%)	0.027	0.868
Preterm birth, *n* (%)	552 (4.9%)	260 (4.6%)	0.633	0.426
Low birth weight, *n* (%)	402 (3.6%)	198 (3.5%)	0.026	0.872
Macrosomia, *n* (%)	728 (6.4%)	393 (7.0%)	1.663	0.197
Small for gestational age, *n* (%)	581 (5.1%)	305 (5.4%)	0.528	0.467
Large for gestational age, *n* (%)	1831 (16.2%)	954 (16.9%)	1.357	0.244
Composite endpoints, *n* (%)	4959 (43.8%)	2510 (44.4%)	0.546	0.460

Abbreviation: BMI, body mass index.

### 3.2 Ranges for Appropriate GWG

#### 3.2.1 Percentile Method

Among the 11,323 pregnant women in the calculation database, 6364 had good maternal and infant outcomes. According to the 25th to 75th percentile distribution of GWG under different pre‐pregnancy weight categories, the ranges for appropriate GWG proportion for pregnant women in the low, normal, and overweight/obese categories of pre‐pregnancy weight were 25.8%–37.8%, 20.3%–30.8%, and 13.0%–23.1%, respectively (Table 3).

**TABLE 3:** Percentile distribution of GWG proportion of pregnant women in training set.

Percentile	P_2.5_	P_5_	P_10_	P_25_	P_50_	P_75_	P_90_	P_95_	P_97.5_
Underweight	13.77%	18.16%	21.01%	25.81%	31.25%	37.76%	43.75%	47.68%	52.27%
Normal weight	10.20%	12.90%	15.74%	20.34%	25.45%	30.77%	35.71%	39.22%	43.08%
Overweight or obesity	2.94%	6.25%	8.70%	13.04%	17.79%	23.08%	28.24%	31.67%	35.20%

#### 3.2.2 OR Method

**FIGURE 2:**
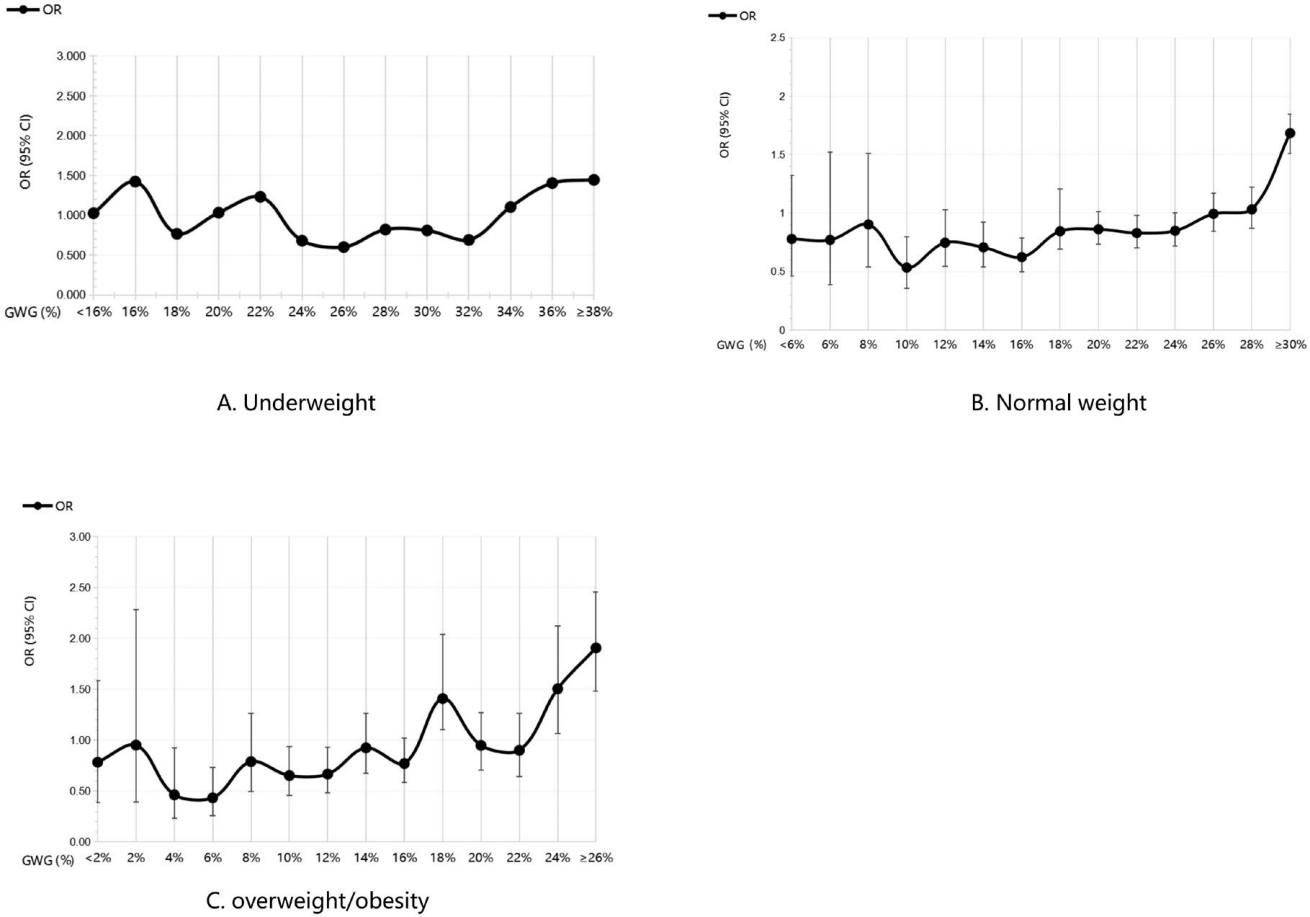
Odds ratio for composite endpoint events by gestational weight gain proportion under Chinese specific BMI categories. Underweight women (A), normal weight women (B), overweight/obese women (C).

For pregnant women in the underweight category of pre‐pregnancy weight, the GWG proportion with OR less than 1 was 24%–34%. Therefore, the range for appropriate GWG proportion for underweight women was 24%–34% (Figure 2A). For pregnant women who had normal weight before pregnancy, when the GWG proportion was 14%–18%, the OR value and the corresponding 95% CI were less than 1. Therefore, 14%–18% was considered to be the range for appropriate GWG proportion for pregnant women with normal weight before pregnancy (Figure 2B). Finally, for pregnant women in the overweight/obese category before pregnancy, even though the OR value was less than 1 when the GWG proportion was less than 18%, its range was too wide. Considering that the corresponding 95% CI was less than 1% when the GWG proportion was in the 4%–8% and 10%–14% ranges, the range for appropriate GWG proportion for overweight or obese women (before pregnancy) was 4% to 14% (Figure 2C).

#### 3.2.3 Combined Risk Curve Method

**FIGURE 3:**
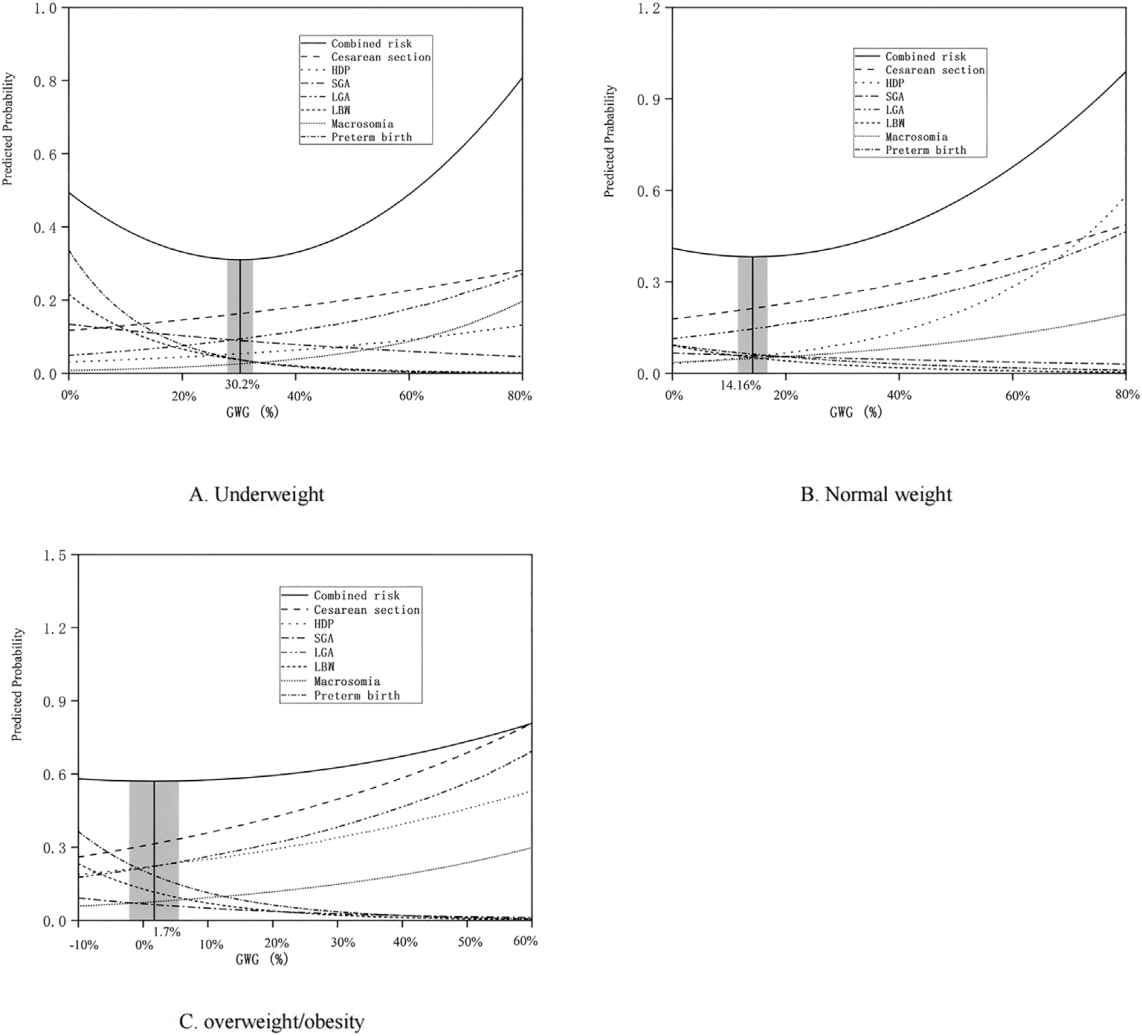
Predicted probabilities of cesarean section, hypertensive disorder complicating pregnancy, small for gestational age, large for gestational age, macrosomia, low birth weight, preterm birth, with increasing gestational weight gain, stratified by Chinese specific BMI categories. Underweight (A), normal weight (B), overweight/obese women (C).

For women who had low weight before pregnancy, the lowest combined predicted probability (*p‐hat* = 0.311) of composite endpoints corresponded to the GWG proportion of 30.2%, and the GWG proportion corresponding to the lowest combined predicted probability increase of no more than 1‰ (*p‐hat* ≤ 0.312) ranged from 27.9% to 32.5% (the interval corresponding to the shaded part in Figure 3A). Meanwhile, for women in the normal weight category before pregnancy, the lowest combined predicted probability (*p‐hat* = 0.383) of composite endpoints corresponded to the GWG proportion of 14.2%, and the GWG proportion corresponding to the lowest combined predicted probability increase of no more than 1‰ (*p‐hat* ≤ 0.384) ranged from 11.5% to 16.8% (the interval corresponding to the shaded part in Figure 3B). For subjects in the overweight/obese category before pregnancy, the lowest combined predicted probability (*p‐hat* = 0.572) of composite endpoints corresponded to the GWG proportion of 1.7%, and the GWG proportion that corresponded to the lowest combined predicted probability increase of no more than 1‰ (*p‐hat* ≤ 0.573) ranged from −2.1% to 5.5% (the interval corresponding to the shaded part in Figure 3C). The equations for the calculations are shown in Appendix 1.

### 3.3 Evaluation of GWG Ranges

In the validation set, the paired chi‐squared test was implemented to statistically compare the GWG proportion ranges obtained for the three weight categories using three different methods. The detection rates of each adverse outcome and composite endpoint event within the recommended reference range were calculated and compared. The rates of detection of adverse maternal and neonatal outcomes of subjects with appropriate weight gain obtained by different methods were compared (Table 4).

**TABLE 4:** Comparison of detection rates of adverse maternal and infant outcomes.

Maternal and neonatal outcomes	OR method	Percentile method	Combined risk curve method
HDP (*n* = 584)	83 (14.2%)	252 (43.1%)	32 (5.4%)
Pre‐eclampsia (*n* = 262)	33 (12.5%)	111 (42.3%)	16 (6.1%)
CS with medical indications (*n* = 1206)	185 (15.3%)	562 (46.6%)	92 (7.6%)
Postpartum hemorrhage (*n* = 314)	46 (14.6)	142 (45.2%)	23 (7.3%)
Preterm birth (*n* = 260)	61 (23.4%)	101 (38.8%)	33 (12.6%)
Low birth weight (*n* = 198)	40 (20.2%)	72 (36.3%)	32 (16.1%)
Macrosomia (*n* = 393)	41 (10.4%)	195 (49.6%)	16 (4.0%)
Small for gestational age (*n* = 305)	56 (18.3%)	139 (45.5%)	28 (9.1%)
Large for gestational age (*n* = 954)	131 (13.7%)	439 (46.0%)	56 (5.8%)
Neonatal asphyxia (*n* = 68)	10 (14.7%)	29 (42.6%)	11 (16.1%)
Composite endpoints (*n* = 2510)	384 (15.2%)	1148 (45.7%)	199 (7.9%)

Abbreviations: CS, cesarean section; HDP, hypertensive disorder complicating pregnancy.

Pregnancy‐induced hypertension, cesarean section with medical indications, pre‐eclampsia, postpartum hemorrhage, premature delivery, LBW infants, macrosomia, SGA, LGA, neonatal asphyxia, and composite endpoint events were the lowest in pregnant women with appropriate GWG calculated by the combined risk curve method. Meanwhile, single adverse maternal and infant outcomes and composite endpoint events were the highest in pregnant women with appropriate GWG obtained by the percentile method (Table 4).

## 4 Discussion

This study found that an inappropriate proportion of GWG was linked to various adverse pregnancy‐related outcomes, while an adequate proportion of GWG was associated with better pregnancy‐related outcomes. The recommended optimal GWG proportion ranges for pregnant women in the low weight, normal weight, and overweight/obese categories were 27.9%–32.5%, 11.5%–16.8%, and −2.1%–5.5%, respectively, according to the Chinese‐specific BMI classification.

GWG is a good indicator of maternal and infant health and pregnancy outcomes. Studies have found that excessive GWG enhances the synthesis of protein and triglycerides, which furthers the risk of adverse pregnancy‐related outcomes, including pregnancy‐induced hypertension, cesarean section, and shoulder dystocia (Rogozińska et al. 2019; Wu et al. 2020). On the other hand, pregnant women with insufficient GWG have insufficient fat reserves and poor nutritional levels, resulting in iron deficiency, anemia, infection, and hyperemesis during pregnancy, which further results in an elevated risk of SGA and premature birth (Goldstein et al. 2018). A GWG value that is appropriate for pregnant women's weight helps attain good delivery outcomes and improves short‐term and long‐term maternal and neonatal health (Sámano et al. 2021; Zhou et al. 2020; Ukah et al. 2019). Attention should thus be paid to health education and nutritional guidance for pregnant women to encourage weight management during pregnancy and improve maternal and infant health.

The proportion of weight gain not only considers GWG but also the basic weight of pregnant women before pregnancy, which provides a corresponding, individualized reference for the judgment of the degree of weight gain during pregnancy. This study calculated the reference value range for appropriate GWG proportion of women in northern China under different pre‐pregnancy BMI classifications. It was found that under the appropriate GWG proportion range, the detection rate of adverse pregnancy and childbirth outcomes was lower, suggesting that GWG proportion, and not GWG alone, may be a more suitable metric of pregnancy weight gain among Chinese women. Using the change of weight gain ratio to judge the weight gain during pregnancy can enable medical personnel to provide pregnancy nutrition management for each pregnant woman in a more targeted and individualized manner. Moreover, in addition to body mass, other physiological indicators of pregnant women, such as uterine height, abdominal circumference, etc., can also reflect the nutritional status of the mother as well as the fetus (Jia et al. 2021; Ross et al. 2020). Such parameters may also be used for nutritional management in pregnant women.

The outcome indicators in this study, including all‐cause cesarean section and gestational diabetes, differ from those used in other studies. All‐cause cesarean section or gestational diabetes has often been included in previous studies when creating GWG range (Zhang et al. 2021; Nagpal et al. 2022; Morisaki et al. 2017; Ota et al. 2011), but these indicators were excluded from the present study. Firstly, the cesarean section rate of Chinese pregnant women has been rising in recent years, ranking first in Asian countries with an incidence of 36.7% in 2018 (Betran et al. 2016; Plows et al. 2018). The elevated rate of cesarean section in China might be associated with various social and psychological reasons, such as pregnant women's apprehension of childbirth pain, concerns about postpartum vaginal laxity, and the insufficient progress of midwifery in certain areas. Thus, substituting cesarean section with medical indications for all‐cause cesarean section can reduce the deviations in the calculation of the appropriate GWG range and increase the rigor of the present study (Betran et al. 2016; Plows et al. 2018). Hence, replacing all‐cause cesarean sections with those medically indicated can reduce the deviations in the calculation of the appropriate GWG range and increase the rigor of the present study.

In addition, the studies which have included gestational diabetes as an outcome have shown that GWG is a protective factor against the incidence rate of gestational diabetes (Zhang et al. 2021; Chuang et al. 2022), whose findings showed that pregnant women who gain excessive weight during pregnancy have a lower risk of gestational diabetes. That is inconsistent with the physiological mechanism of gestation (Plows et al. 2018; Johns et al. 2018; McIntyre et al. 2019). This is possibly due to the fact that the diagnosis time of gestational diabetes is usually the second trimester, and those pregnant women diagnosed with gestational diabetes follow the doctor's advice to scientifically control their weight, causing their overall GWG to be less than that of pregnant women not diagnosed with gestational diabetes (Chuang et al. 2022; Barakat et al. 2019; Brown et al. 2017; Rasmussen et al. 2020). And given that GDM is mainly a metabolic disease, there are many risk factors. In addition to excessive GWG during pregnancy, pre‐pregnancy weight, a low vitamin D level, increased fat consumption, genetic factors, psychological stress, and negative mood etc. are risk factors for GDM (Mahendra and Fall 2021; Spaight et al. 2016). Therefore, gestational diabetes was excluded from the outcomes to increase the accuracy of the selection of outcome indicators in this study. In the end, there are many elements that may affect the range for appropriate GWG, which is why the selection of outcome indicators needs to be considered more comprehensively.

The present study has several advantages. Firstly, three different methods were employed to calculate the suitable weight gain range for pregnant women. The ranges obtained were preliminarily verified and evaluated, which comprehensively showed the weight gain range under different methods and revealed the differences and similarities among different statistical methods. Secondly, the research design of the training set and validation set greatly increased the credibility of the research results. Not only can the GWG range recommended in the present study serve as a reference for clinicians to manage the weight of pregnant women, but it can also add to the pregnancy healthcare knowledge for pregnant women and their families, bridge the psychological gap between medical workers and patients, and help to build a better doctor–patient relationship. Additionally, it is worth noting that this research may serve as a valuable reference for authoritative institutions in China in the formulation of evidence‐based guidelines for weight gain during pregnancy.

At the same time, it is crucial to consider the limitations of this study. First, the present work is a single center study, which restricts the generalizability of the research results to a certain extent. As such, the appropriateness of extrapolation of the conclusions of this work should be confirmed through future studies. Second, the range of GWG proportion recommended in this study is slightly narrow, which shows that the establishment of a range for appropriate GWG needs further exploration and integration of various methods. Third, the pre‐pregnancy BMI classification adopted in this study is based on the BMI classification standard recommended by the WGOC, which is not a classification standard specific to Chinese women of childbearing age. Therefore, the BMI classification standard of Chinese women of childbearing age needs to be further optimized on the basis of the Chinese adult BMI standard.

Given the limitations of this study, its findings cannot be directly adopted as a nationwide guideline. However, this research may serve as a valuable reference for authoritative institutions in China in the formulation of evidence‐based guidelines for weight gain during pregnancy.

## 5 Conclusions

The GWG proportion takes into account the pre‐pregnancy weight level, which helps clinicians tailor weight gain recommendations to the individual needs of pregnant women. The appropriate GWG proportion for pregnant Chinese women was found to be in the ranges of 27.9%–32.5% for underweight, 11.5%–16.8% for normal weight, and −2.1%–5.5% for overweight/obese pregnant women. The findings of this study may offer valuable insights for the refinement of nutrition guidelines and public health policies related to pregnancy, particularly in urban women.

## Author Contributions

**Yin Jia:** conceptualization (supporting), formal analysis (lead), software (lead), writing – original draft (equal), writing – review and editing (equal). **Haili Jiang:** conceptualization (equal), investigation (equal), methodology (equal), project administration (equal), resources (lead), supervision (equal), writing – original draft (equal). **Yang Yu:** funding acquisition (supporting), methodology (equal), supervision (equal), visualization (equal). **Yuhui Fu:** data curation (equal), investigation (equal), resources (equal). **Yue Li:** data curation (equal), investigation (equal), resources (equal). **Huili Wang:** conceptualization (lead), funding acquisition (lead), methodology (equal), supervision (lead).

## Conflicts of Interest

The authors declare no conflicts of interest.

## Data Availability

The data that support the findings of this study are available on reasonable request from the corresponding author. The data are not publicly available due to privacy or ethical restrictions.

## Appendix 1

Exponential function model‐derived optimal GWG proportions corresponding to the lowest predicted probability of adverse pregnancy outcomes across pre‐pregnancy BMI categories.

**Table:**

	Combined risk	Optimal GWG proportion (lowest value)
Underweight	*Y* = 2.008431*x* ^2^ − 1.213009*x* + 0.493810	27.92%–32.48% (30.20%)
Normal weight	*Y* = 1.402246*x* ^2^ − 0.396982*x* + 0.410441	11.54%–16.77% (14.16%)
Overweight/obesity	*Y* = 0.697044*x* ^2^ − 0.023711*x* + 0.571076	−2.14%–5.54% (1.70%)
